# Characterization of the Variability of Epstein-Barr Virus Genes in Nasopharyngeal Biopsies: Potential Predictors for Carcinoma Progression

**DOI:** 10.1371/journal.pone.0153498

**Published:** 2016-04-12

**Authors:** Ana V. Banko, Ivana B. Lazarevic, Miljan M. Folic, Vojko B. Djukic, Andja M. Cirkovic, Danijela Z. Karalic, Maja D. Cupic, Tanja P. Jovanovic

**Affiliations:** 1 Institute of Microbiology and Immunology, Faculty of Medicine, University of Belgrade, Belgrade, Serbia; 2 Clinic of Otorhinolaryngology and Maxillofacial Surgery, Clinical Center of Serbia, Faculty of Medicine, University of Belgrade, Belgrade, Serbia; 3 Institute for Medical Statistics and Informatics, Faculty of Medicine, University of Belgrade, Belgrade, Serbia; The University of North Carolina at Chapel Hill, UNITED STATES

## Abstract

Epstein-Barr virus (EBV) infection is a significant factor in the pathogenesis of nasopharyngeal carcinoma, especially in the undifferentiated carcinoma of nasopharyngeal type (UCNT, World Health Organization type III), which is the dominant histopathological type in high-risk areas. The major EBV oncogene is latent membrane protein 1 (LMP1). LMP1 gene shows variability with different tumorigenic and immunogenic potentials. EBV nuclear antigen 1 (EBNA1) regulates progression of EBV-related tumors; however, the influence of EBNA1 sequence variability on tumor pathogenesis is controversial. The aims of this study were to characterize polymorphisms of EBV genes in non-endemic nasopharyngeal carcinoma biopsies and to investigate potential sequence patterns that correlate with the clinical presentation of nasopharyngeal carcinoma. In total, 116 tumor biopsies of undifferentiated carcinoma of nasopharyngeal type (UCNT), collected from 2008 to 2014, were evaluated in this study. The genes *EBNA2*, *LMP1*, and *EBNA1* were amplified using nested-PCR. *EBNA2* genotyping was performed by visualization of PCR products using gel electrophoresis. Investigation of *LMP1* and *EBNA1* included sequence, phylogenetic, and statistical analyses. The presence of EBV DNA was significantly distributed between TNM stages. *LMP1* variability showed six variants, with the detection of the first China1 and North Carolina variants in European nasopharyngeal carcinoma biopsies. Newly discovered variants Srb1 and Srb2 were UCNT-specific *LMP1* polymorphisms. The B95-8 and North Carolina variants are possible predictors for favorable TNM stages. In contrast, deletions in *LMP1* are possible risk factors for the most disfavorable TNM stage, independent of *EBNA2* or *EBNA1* variability. A newly discovered *EBNA1* subvariant, P-thr-sv-5, could be a potential diagnostic marker, as it represented a UCNT-specific *EBNA1* subvariant. A particular combination of *EBNA2*, *LMP1*, and *EBNA1* polymorphisms, *type 1/Med/P-thr* was identified as a possible risk factor for TNM stage IVB or progression to the N3 stage.

## Introduction

Nasopharyngeal carcinoma (NPC) is an aggressive human malignancy that originates from the epithelial cells of the retronasal cavity. It is rare in most populations around the world with an incidence below 1 per 100 000 persons per year in Europe and the USA; however, in southern China and southeast Asia, NPC is endemic, with an incidence rate of 20–30 per 100 000 persons per year [1]. The undifferentiated carcinoma of nasopharyngeal type (UCNT, World Health Organization type III) is the dominant histopathological type in high-risk areas. The remarkable geographic variations in NPC prevalence are the result of the complex development of this carcinoma [2]. It includes interaction between environmental carcinogens (food, tobacco smoke, alcohol consumption, inhalant, and Epstein-Barr virus infection) and genetic predisposition based on HLA (human leukocyte antigen) polymorphisms and chromosomal 3p LOH (loss of heterozygosity) [3]. This theory is supported by NPC clustering in families from diverse populations [4].

Epstein-Barr virus (EBV) infection is a key environmental factor of UCNT, and is classified as a group 1 carcinogenic agent by the International Agency for Research and Cancer (IARC). In endemic regions, UCNT is almost universally associated with EBV infection. NPC usually has type 2 EBV latency with EBNA1 driven by the Qp promoter, expression of EBER (EBV encoded RNA) and BARTs (BamHI A rightward transcripts), LMP2 and variable expression of LMP1 [5]. The establishment of latent transforming infection in an epithelial cell together with genetic changes that may facilitate latent infection or are synergistic with EBV transforming proteins are likely to be the crucial steps in the development of NPC. Although EBV is highly prevalent in the human population, there are still unidentified genome specificities that contribute to pathogenesis of NPC. On the other hand, geographically associated EBV gene polymorphisms in endemic regions are well known.

EBV is classified as type 1 or 2, mainly based on the divergence within the EBV nuclear antigen 2 (EBNA2) gene, which encodes an essential protein in the EBV transformation process of B lymphocytes [6]. Geographical distributions of genotypes show the dominance of EBV type 1, especially in Europe, Asia, and North and South America. The association between genotype and disease has not yet been established [7].

Latent membrane protein 1 (LMP1) is a crucial EBV oncogene, which has been shown to transform rodent fibroblasts *in vitro* and induce tumors in nude mice [8,9]. The transformation and immortalization of B lymphocytes occur by inducing B-cell activation markers and expression of the anti-apoptotic *A20* and *bcl-2* genes [10]. The oncogenic potential of LMP1, which results in B cell transformation, is suggested by its high functional similarity to the tumor necrosis factor receptor (TNFR) family members, CD40 and TNFR1 [11].

The C-terminal region of LMP1 is significantly heterogeneous. Seven LMP1 strains have been defined based on nucleotide sequence variations: Alaskan (AL), China1, China2, China3, Mediterranean with (Med+) or without (Med−) deletions, and North Carolina (NC) [12,13]. These variants are distinguished by the presence or absence of a 30-bp deletion, the number of characteristic 11-amino acid (33-bp) repeats, and defined nucleotide and amino acid changes in comparison with the prototype sequence, B95-8 [13]. It has been suggested that some LMP1 variants have potentially higher tumorigenic activity and lower immunogenic potential of EBV [14]. This concept refers to the presence of the 30-bp deletion [15], which also has the capability to prolong the half-life of the LMP1 protein [3,16]. In addition, a geographically specific distribution of LMP1 variants has been described [17].

*EBNA1* is the only EBV gene expressed in all infected cells. Thus, it may play a critical role in the onset, progression, and persistency of EBV-related tumors. There have been reports about the anti-apoptotic properties of EBNA1 in Burkitt’s lymphoma, inhibiting p53-dependent apoptosis [18]. It is also known that EBNA1 is essential for virus replication, maintenance of extrachromosomal episomes, and transcriptional control of the viral latency programs, through sequence-specific binding to its replication origin, OriP. The Gly-Ala repeats in the sequence were initially reported to prevent the presentation of EBNA1 on major histocompatibility complex (MHC) class I molecules and preclude recognition by CD8^+^ cytotoxic T lymphocytes [19]. More recent studies indicate that the dominant role of the mentioned repeats is to reduce the translational efficiency of EBNA1 and to inhibit the initiation of translation. Those mechanisms result in fewer EBNA1 peptides expressed on the cell surface and in less efficient recognition by EBV-specific CD8^+^ T cells [5].

EBNA1 sequence variability is classified into five subtypes: two prototype sequences P-ala (B95-8 prototype) and P-thr, and three variant sequences V-val, V-leu, and V-pro. The subtype V-ala has been added afterwards [17]. Subtypes are identified according to the amino acid present in locus 487 [20], and sub-variants based on amino acid substitutions on loci other than locus 487. The literature data about the association between tumor status and EBNA1 sequence variability are controversial [21,22]. However, the geographically specific distribution of EBNA1 subtypes is unambiguous.

Serbia and the countries in Southeast Europe are considered non-endemic regions for NPC with a dominance of the UCNT type. Reports about EBV variability are very rare [23]. However, thus far, no data are available on the association between EBV gene polymorphisms and clinical characteristics of cancer. The aims of the present study are to characterize EBV gene polymorphisms circulating in NPC isolates from this geographic region and to investigate potential sequence patterns that correlate with NPC clinical presentation. According to proposed, the results of EBV variability also demonstrated newly variants. In addition, risk factors for favorable and disfavorable TNM stages were identified. The majority of them were identified for the first time.

## Materials and Methods

### Patients and samples

This study consists of tumor biopsies collected from 116 patients between 2008 and 2014 with histologically confirmed UCNT. Each tissue sample was obtained by incisional biopsy during endoscopy of nasopharynx. Archived tissue blocks fixed in formalin and embedded in paraffin were retrieved from the Clinic of Otorhinolaryngology and Maxillofacial Surgery, Clinical Center of Serbia. Sample collection and research were approved by the Ethics Committee of the Faculty of Medicine, University of Belgrade, No.29/VI-12. As sample collection was retrospective, both institutions waived the need for written informed consent from the donors (review board of the Clinic of Otorhinolaryngology and Maxillofacial Surgery, Clinical Center of Serbia, number No.1419 and Ethics Committee of the Faculty of Medicine, University of Belgrade, number No.29/XI-8).

All samples were collected from Caucasian individuals. Eighty-five patients were male (73.3%) and 31 were female (26.7%). The average age was 54 ± 13.1 years (18 to 78). After diagnosis by endoscopic biopsy, 63 patients were initially treated with chemotherapy (CT), 31 with chemo-radiotherapy (CRT), 9 with radiotherapy (RT), 7 surgically, and 6 only symptomatically. According to the accessible data, all patients were classified by five different criteria: sex (male, female); tobacco smoking (57 smokers and 24 non-smokers); the history of any illness (65 positive and 44 negative); TNM staging by the American Joint Committee on Cancer (15 with stage Tx, 4 at stage I, 30 at stage II, 28 at stage III, 9 at stage IVA, 27 at stage IVB, and 3 at stage IVC); and the last known outcome of disease (4 with therapy in progress, 12 with complete remission, 7 with partial regression, 6 with stabilization, and 32 with progression and/or metastasis of the tumor).

### Deparaffinization and DNA isolation

Three 10-mm-thick tissue sections from each block were placed in a sterile, plastic 1.5-ml PCR tube, deparaffinized with xylene, rehydrated in alcohol, and then air-dried. The tissue sections were then resuspended and lysed overnight at 56°C in 180 μl digestion buffer (QIAGEN, Hilden, Germany) and 20 μl proteinase K (QIAGEN, Hilden, Germany). Viral DNA was isolated using a QIAamp Mini Kit (QIAGEN, Hilden, Germany), according to the manufacturer’s instructions.

### EBV typing by EBNA2

EBV typing was performed in 32 EBNA2-positive biopsies, by nested-PCR, as previously described, using primers that were reported by Mendes et al. [23,24]. The first reaction amplified a common 596-bp region covering almost the entire EBNA2 extent, followed by two separate nested reactions amplifying distinctive regions of 497 bp for EBV type 1 and 150 bp for EBV type 2. EBV types 1 and 2 were distinguished by identifying either the 497-bp fragment or the 150-bp fragment in gel electrophoresis.

### LMP1 carboxy-terminal region sequencing

Amplification of the C-terminus of the LMP1 gene was performed by nested-PCR as described previously, using primers that were reported by Li et al. [23,25]. Thirty-five LMP1-positive PCR products were purified using a QIAGEN MinElute Purification Kit (QIAGEN, Hilden, Germany), according to the manufacturer’s instructions. For cycle sequencing reactions, internal PCR primers and a Big Dye Terminator v 3.1 Cycle Sequencing Kit (Applied Biosystems, Foster City, CA, USA) were used. Sequencing was carried out in an automatic sequencer (ABI PRISM 310 Genetic Analyzer; Applied Biosystems, Foster City, CA, USA). Both sense and antisense strands were sequenced and compared.

### EBNA1 carboxy-terminal region sequencing

Amplification of the C terminus of the EBNA1 gene was performed by nested-PCR using primers reported by Lorenzetti et al. [26]. Both PCR reactions were carried out in 40 cycles, the first reaction at 95°C for 1 min, 57°C for 2 min, and 72°C for 90 sec; and the second reaction at 95°C for 1 min, 60°C for 2 min, and 72°C for 90 sec. After analysis of PCR products by gel electrophoresis with ethidium bromide staining, 40 EBNA1-positive products were purified, used in cycle sequencing reactions, and sequenced based on the same principles described for LMP1 sequencing.

### Sequence and phylogenetic analysis

The 506-bp and 329-bp nucleotide sequences of LMP1 and EBNA1, respectively, were separately aligned and compared with a reference wildtype sequence in Bioedit 7.0.5.3 software [27]. Using the same software, we searched for characteristic amino acid changes described by Edwards et al. [13] in order to identify and classify LMP1 variants. In addition, we classified EBNA1 subtypes and sub-variants after inspecting signature amino acid changes at the following positions: 471, 475, 476, 479, 487, 492, 499, 500, 502, 517, 520, 524, 525, 528, and 533.

For representative and reference LMP1 sequences, 13 sequences obtained from the GenBank/EMBL7DDBJ database under the accession numbers V01555, AY493742, AY493743, AY337721, AY337722, AY493810, AY337723, AY493835, AY337724, AY493799, AY337725, AY337726, and X58140 were used. For representative and reference EBNA1 sequences, 10 sequences obtained from the GenBank/EMBL7DDBJ database under the accession numbers V01555, GU475455, JN986939, AF192742, GU475448, AF192743, GU475431, AF192744, JN986947, and GU475442 were used. Thirty-five LMP1 and 40 EBNA1 NPC sequences from this study are available in the GenBank/EMBL7DDBJ database with accession numbers: JF901794-JF901802, JN971085-JN971091, and KT820429-KT820488. The LMP1 and EBNA1 sequences identified were aligned pairwise using the ClustalW method implemented in the MEGA 6.0 software [28]. Adequate reference sequences from the GenBank/EMBL/DDBJ database were used in both alignments. The most appropriate models for evolution for C-terminal regions of LMP1 and EBNA1 genes were inferred using jModelTest 2.1.4 [29]. Maximum-likelihood trees were estimated according to the defined best-fit F81+I+G evolutionary model by using the PhyML 3.0 software [30]. Statistical significance of phylogeny was estimated by bootstrap analysis with 1,000 pseudo-replicate datasets. Graphical presentation and edition of phylogenetic trees were performed with Fig Tree 1.4.0 [31] and MEGA 6.0 [28] software.

### Statistical analyses

The chi-squared or Fisher's exact test and Student’s *t*-test were used for statistical analysis. To investigate potential risk factors or predictors of disease, statistical testing was followed by univariate logistic regression analysis. Analyses were performed by SPSS v.21 for Windows (SPSS Inc., Chicago, IL, USA). *P*-value ≤ 0.05 was considered statistically significant.

## Results

### EBV typing

The frequencies of EBV type 1 or type 2 were determined from 32 EBNA2 isolates. EBV type 1 was present in 93.75% of the samples (30/32) and EBV type 2 in 6.25% of the samples (2/32).

### LMP1 variant characterization and sequence analysis

Thirty-five sequences of the EBV *LMP1* gene were obtained, analyzed, and compared with the B95-8 prototype sequence. Characteristic nucleotide variability including variant characterization, detection of deletions, determination of the number of 11-amino acid repeats, and inspection of amino acid changes were investigated, followed by phylogenetic analysis.

**Fig 1:**
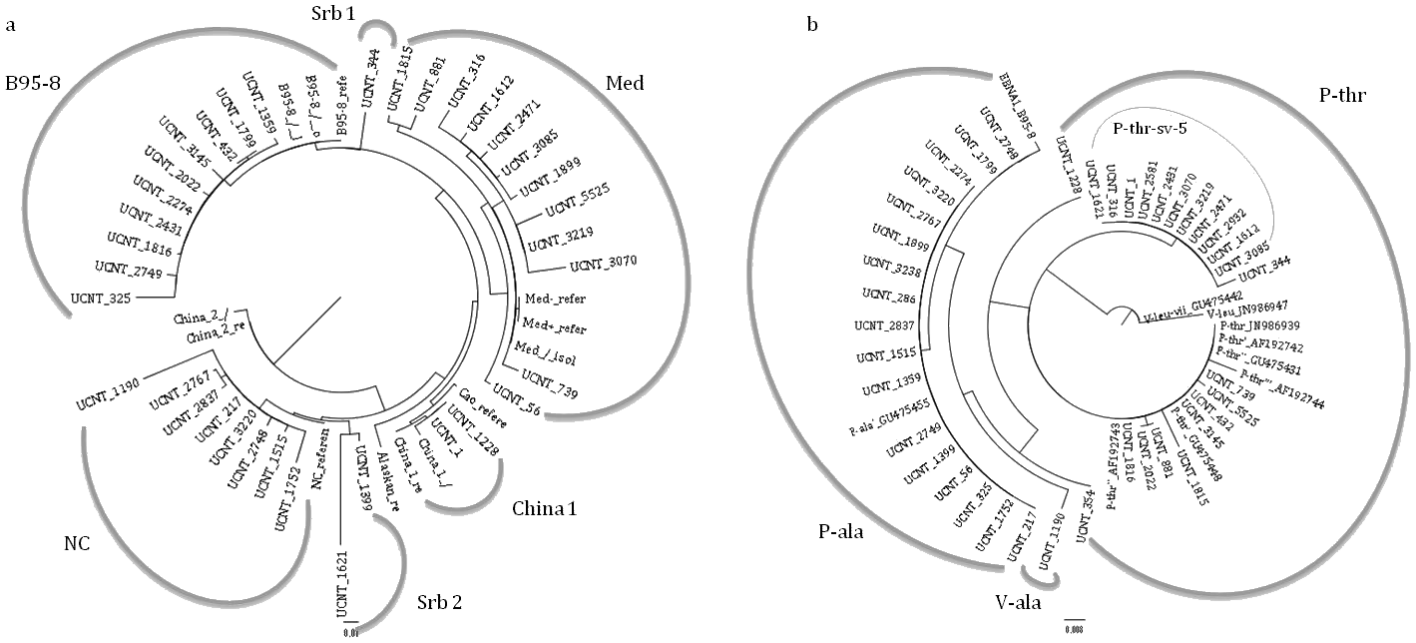
Phylogenetic trees of the C-termini of LMP1 and EBNA1. (a) Thirty five 506-bp fragments of LMP1 (from coordinates 168719–168213) NPC sequences available in GenBank/EMBL7DDBJ database with accession numbers: JF901794-JF901802, JN971085-JN971091 and KT820429-KT820448 and 13 sequences obtained from GenBank/EMBL7DDBJ database under the following accession numbers: V01555, AY493742, AY493743, AY337721, AY337722, AY493810, AY337723, AY493835, AY337724, AY493799, AY337725, AY337726 and X58140. (b) Forty 329-bp fragments of EBNA1 (from coordinates 109261–109590) NPC sequences available in GenBank/EMBL7DDBJ database with accession numbers KT820449-KT820488 and 10 sequences obtained from GenBank/EMBL7DDBJ database under the following accession numbers: V01555, GU475455, JN986939, AF192742, GU475448, AF192743, GU475431, AF192744, JN986947 and GU475442.

As shown in Fig 1, the phylogenetic analysis clustered *LMP1* sequences from this study, along with other isolates from GenBank, into four groups. The groups are defined by 4 of 7 known *LMP1* variants, namely B95-8, Med, China1, and NC. In addition, inspection of variant-characteristic amino acid changes defined by Edwards et al. was used to confirm the phylogenetic grouping of the sequences [13]. The most dominant variant was Med (34.3%) (Table 1). However, three LMP1 isolates did not match any of the variants from the above-mentioned classifications and were shown as two extra branches in the phylogenetic tree. In keeping with the previous nomenclature, where the variants were named after the geographic location where they were first isolated, the two new variants from this study were temporarily named Serbia1 (Srb1) for isolate UCNT344, and Serbia2 (Srb2) for isolates UCNT1399 and UCNT1621.

**Table 1:** Distribution of three LMP1 characteristics and EBNA1 subtypes in EBV isolates from NPC biopsies.

	EBNA1 subtype			LMP1 variant	Number of LMP1 33-bp tandem repeat units		LMP1 deletion	
	P-thr	P-ala	V-ala		2–4.5	5–6		
	5	5	-	B95-8	10	-	No del	10
	2	-	-	China 1	-	2	30-bp-del	2
	0	7	1	NC	6	2	No del	8
	1	-	-	Med	1	-	30-bp-del	1
	4	1	-	Med	1	4	69-bp-del	5
	5	1	-	Med	3	3	No del	6
	1	-	-	Srb 1	-	1	30-bp-del	1
	1	-	-	Srb 2	-	1	69-bp-del	1
	-	1	-	Srb 2	1	-	No del	1
*P*-values				0.004^1^				
					0.002^2^			
	0.003^3^							
	0.024^4^							

^1^*P*-value denoting significant differences in the distribution of 33-bp repeats (≤ 4.5 or > 4.5) among *LMP1* variants. In all B95-8 and in majority of the NC isolates, the number of repeats was ≤ 4.5, in contrast to all China1 and Srb1 isolates with > 4.5 repeats.

^2^*P*-value denoting significant differences in the distribution of 33-bp repeats (≤ 4.5 or > 4.5) between non-deleted and deleted isolates. The majority of non-deleted isolates (80%) had ≤ 4.5 repeats, in contrast to the majority of deleted isolates (80%), which had > 4.5 repeats.

^3^*P*-value denoting significant differences in the distribution of *LMP1* variants among EBNA1 subtypes.

^4^*P*-value denoting significant differences in the presence of *LMP1* deletions among EBNA1 subtypes.

From nucleotide deletion analyses of *LMP1* sequences, it was determined that most isolates (71.4%) did not include deletions. However, two deletions were identified in almost one third of all sequences (28.6%): the specific 10-amino acid/30-bp deletion (spanning codons 346–355), which was found in four isolates (11.4%), and a rare 23-amino acid/69-bp deletion (spanning codons 333–355), which was found in six isolates (17.2%) (Table 1).

It has been shown that the C-terminal domain of LMP1 could contain various numbers of 11-amino acid repeats located between amino acids 250 and 308 [32]. The B95-8 prototype sequence has four perfect repeats with a disruption of 5 amino acids between the second and the third repeat (4.5 11-amino acid repeats). Therefore, isolates from this study were classified into two groups: those with 4.5 repeats or less, and those with more than 4.5 repeats (Table 1). The number of repeats varied from two to six, and the group with 4.5 repeats or less was the most common (62.9%).

**Fig 2:**
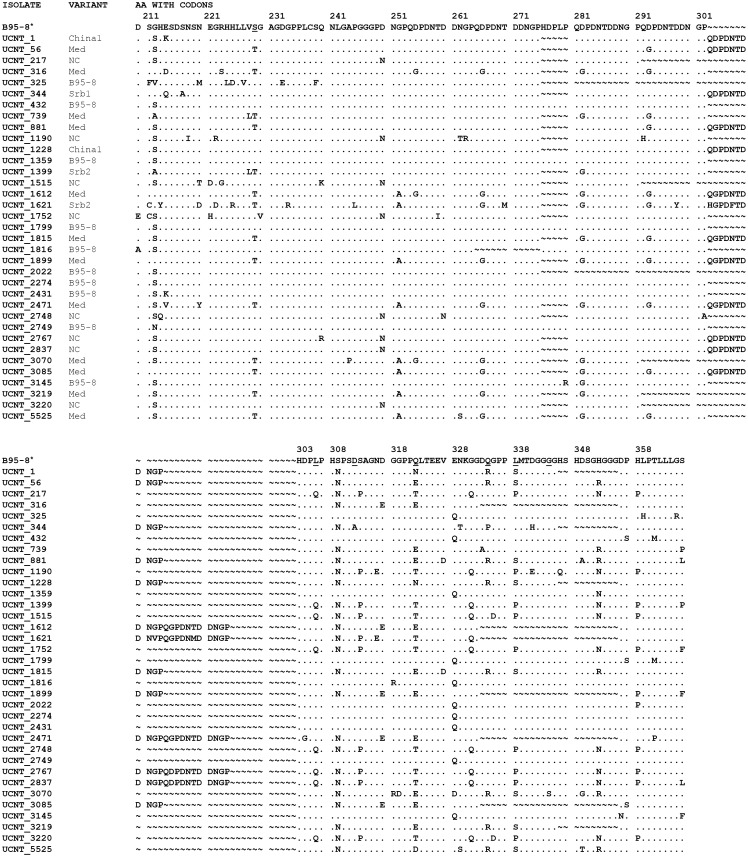
Alignment of obtained LMP1 sequences isolated from UCNT biopsies. B95-8* represents aa sequence of the prototype LMP1. Seven characteristic aa positions for variant discrimination, described by Edwards et al. (1999) are underlined. China1 and NC isolates showed additional representative aa changes which were not listed in known classification: China1 (position 322 and 338) and NC (position 338). Of 85 aa substitutions which were identified at additional 58 positions, several were unique for single variant: H→R at 352 for Med-, E→Q at 328 and S at 309 for B95-8, and D→N at 250 for NC.

To complete the sequence characterization of LMP1 isolates from this study, it was necessary to identify the amino acid changes. In the first step, the analysis included seven characteristic amino acid positions for variant discrimination, described by Edwards et al. (Fig 2) [13]. Moreover, 85 amino acid substitutions were identified at an additional 58 positions and some of them were unique for specific variants (Fig 2).

### EBNA1 sequence variation

Forty sequences of *EBNA1* were obtained, analyzed, and compared with the B95-8 prototype sequence. According to the amino acid substitutions and clustering of isolates in the phylogenetic tree, three subtypes were identified: two prototype subtypes P-ala and P-thr and one variant subtype V-ala (Fig 1). The most frequent subtype was P-thr (55%) (Table 2). Investigations of characteristic nucleotide variability aside from subtype-specific amino acid substitutions included subvariant characterization within the scope of each subtype. Each identified subvariant (sv) (P-ala-sv-1 and -2, P-thr-sv-2, -4, -5, and -6, and V-ala-sv-1), clustered separately in the phylogenetic tree and had representative amino acid substitutions (Table 2).

**Table 2:** EBNA1 C-terminal nucleotide and amino acid changes found in three subtypes and seven subvariants identified in this study.

	B95-8^1^ (P-ala)	P-ala- sv-1	P-ala- sv-2	P-thr	P-thr-sv-2^2^	P-thr-sv-4^2^	P-thr-sv-5^2^	P-thr-sv-6^2^	V-ala- sv-1^2^
Locus									
**476**	CCG (Pro)			CAG (Gln)			CAG (Gln)	CAG (Gln)	
**483**	GAA (Glu)					GAC (Asp)	GAC (Asp)	GAC (Asp)	GAC (Asp)
**487**	GCT (Ala)			ACT (Thr)	ACT (Thr)	ACT (Thr)	ACT (Thr)	ACT (Thr)	
**492**	AGT (Ser)			TGT (Cys)	TGT (Cys)		TGT (Cys)	TGT (Cys)	
**499**	GAC (Asp)	GAA (Glu)	GAA (Glu)	GAT (Asp)	GAT (Asp)	GAT (Asp)	GAT (Asp)	GAT (Asp)	GAG (Glu)
**502**	ACT (Thr)							ATT (Ile)	AAT (Asn)
**520**	CTA (Leu)			CTC (Leu)	CTC (Leu)		CTC (Leu)	CTC (Leu)	CTC (Leu)
**524**	ACT (Thr)	ATT (Ile)	GTT (Val)	ATT (Ile)	ATT (Ile)		ATT (Ile)	ATT (Ile)	ATT (Ile)
**529**	CCA (Pro)								CAA (Gln)
**Number of isolates**	**-**	**2**	**15**	**8**	**1**	**1**	**11**	**1**	**1**
**Total**	**17 (42.5%)**			**22 (55%)**					**1 (2.5%)**

^1^Prototype sequence (represents the P-ala subtype)

^2^New subvariants: P-thr-sv-2, P-thr-sv-4, P-thr-sv-5, and P-thr-sv-6.

### Correlation between polymorphisms in the *EBNA2*, *LMP1*, and *EBNA1* genes

Investigation of any association between three specific *LMP1* sequence characteristics (variant, deletions, and the presence of ≤ 4.5 or > 4.5 33-bp repeats) resulted in two statistical significances (Table 1). Significant differences were found in the distribution of the number of 33-bp repeats (≤ 4.5 or > 4.5) between *LMP1* variants (*P* = 0.004), and between non-deleted and deleted isolates (*P* = 0.002) (Table 1).

Statistical analysis did not show any correlation between different *LMP1* sequence variabilities and *EBNA2* genotypes. However, it was found that *LMP1* variants had significant differences in distribution between EBNA1 subtypes (*P* = 0.003), and that the presence of *LMP1* deletions had significant differences in distribution between *EBNA1* subtypes (*P* = 0.024) (Table 1).

### Correlation between polymorphisms of EBV genes and clinical parameters

Investigation of the correlation between the presence of EBV DNA and accessible anamnestic and clinical data showed significant differences in distribution between TNM stages (*P* = 0.029). In stage Tx, where the primary tumor could not be evaluated (15/116), EBV DNA was not detected. All combinations of TNM stages that were found in UCNT biopsies are summarized in Table 3.

**Table 3:** Combinations of TNM stages found in UCNT biopsies.

TNM staging	EBNA2-positive biopsies	LMP1-positive biopsies	EBNA1-positive biopsies	EBV DNA-negative biopsies
TxN1M0	-	-	-	8
TxN2M0	-	-	-	2
TxN3M0	-	-	-	1
TxN3aM0	-	-	-	3
TxN3bM0	1	1	1	1
T1N0M0	2	2	2	2
T1N1M0	6	6	7	7
T1N2M0	4	5	5	3
T1N3aM0	2	2	2	8
T1N3aM1	-	-	-	1
T1N3bM0	-	-	-	2
T2N0M0	1	2	2	3
T2N1M0	2	4	4	7
T2N2M0	3	3	3	1
T2N2M1	1	-	1	-
T2N3M0	-	-	-	1
T2N3aM0	-	-	-	5
T2N3bM0	1	1	1	-
T3N0M0	-	1	1	5
T3N1M0	1	2	2	3
T3N2M0	2	1	2	3
T3N3M0	1	1	1	-
T3N3aM0	2	2	3	-
T4N0M0	-	-	-	6
T4N1M0	2	2	2	-
T4N2M0	1	-	1	-
T4N2M1	-	-	-	1
T4N3aM0	-	-	-	3
**Total**	32	35	40	76

Two types of disease outcomes were defined to ascertain eventual EBV predictors and risk factors for UCNT pathogenesis. The first type referred to the last known clinical outcome and the second type referred to TNM stage. Among the EBV gene sequence characteristics identified, there were no potential predictors for any clinical outcome, even when they were grouped differently. Furthermore, considering TNM stages (TNM stage I to IVB), the risk factors had not been shown either. However, when the most disfavorable UCNT stage of EBV positive patients, TNM stage IV, was separated from stages TNM stages I-III, the *LMP1* variants B95-8 and NC together have been identified as possible predictors for tumor without intracranial extension, TNM stages I-III (*P* = 0.055). Moreover, when TNM stages were grouped as TNM stages I-IVA and TNM IVB, as the most disfavorable UCNT stage of EBV positive patients, the presence of *LMP1* deletion had been identified as a possible predictor for TNM IVB (*P* = 0.012) (Table 4).

**Table 4:** Distribution of LMP1 deletions and different combinations of EBV gene polymorphisms between TNM stages I-IVA and TNM stage IVB found in UCNT biopsies.

	TNM I—IVA	TNM IVB	total	*P*-value^1^	*P*-value^2^
**The presence of LMP1 deletion**					
Non-deleted LMP1	23	2	25		***P* = 0.012**
Deleted LMP1	5	5	10		
**EBNA2 type/LMP1 variant**					
Type 1/B95-8	7	-	7	***P* = 0.016**	
Type 1/Srb 1	-	1	1		
Type 1/Med	4	4	8		
Type 1/NC	7	-	7		
Type 1/Srb 2	1	1	2		
Type 2/Med	1	-	1		
Type 2/NC	1	-	1		
**LMP1 variant/ EBNA1 subtype**					
B95-8/P-thr	5	-	5	***P* = 0.042**	
B95-8/P-ala	5	-	5		
Srb 1/P-thr	-	1	1		
Med/P-thr	6	4	10		
Med/P-ala	2	-	2		
China 1/P-thr	1	1	2		
NC/P-ala	7	-	7		
NC/V-ala	1	-	1		
Srb 2/P-thr	1	-	1		
Srb 2/P-ala	-	1	1		
**deleted or non-deleted LMP1/EBNA1 subtype**					
deleted/P-thr	4	5	9	***P* = 0.049**	
deleted/P-ala	1	-	1		
Non-deleted/P-thr	10	1	11		
Non-deleted/P-ala	12	1	13		
Non-deleted/V-ala	1	-	1		
**EBNA2 type/LMP1 variant/EBNA1 subtype**					
*type 1/Med/P-thr*	2	4	6		***P* = 0.013**
The other appearances of this polymorphism	19	1	20		

^1^*P*-value denoting significant differences in the distribution of different EBV polymorphisms between the grouped TNM stages

^2^*P*-value for possible predictors for TNM stage IVB

According to statistical results, the most prominent and specific LMP1 amino acid substitutions from Serbian isolates (212, 229, 250, 309, 317, 322, 328, and 399) had not been shown as potential risk factors for TNM stage evolution or progression as a clinical outcome.

To assess potential associations between clinical outcome and comprehensive variability of three EBV genes, ten different EBV polymorphisms were defined. Each polymorphism comprised of characteristics of two or three EBV genes. Among appearances of the major polymorphisms, which were defined by EBNA2 genotype, LMP1 variant, and EBNA1 subtype, the most frequent polymorphism was *type 1/Med/P-thr* (26.9%). Considering the previous grouping of TNM stages as two pathohistological outcomes (TNM stages I-IVA and TNM stage IVB), *type 1/Med/P-thr* was identified as possible risk factor for TNM stage IVB (*P* = 0.013) (Table 4). Statistical analyses also showed significant differences in distribution of diverse appearances of three defined polymorphisms between the two groups of TNM stages: EBNA2 genotype/LMP1 variant (*P* = 0.016), LMP1 variant/EBNA1 subtype (*P* = 0.042), and deleted or non-deleted LMP1/EBNA1 subtype (*P* = 0.049) (Table 4).

## Discussion

Although the role of the variability of EBV genes in pathogenesis of nasopharyngeal carcinoma was widely investigated in non-endemic and endemic regions, the literature data concerning the correlation between the genetic variability of EBV genes and clinical aspects of NPC were provided only by researchers from endemic regions such as Malaysia and Thailand [33,34].

The presence of EBV DNA in UCNT tissue did not correlate with any anamnestic or clinical data, except for TNM stages. Particularly, the frequency of EBV DNA-positive biopsies slightly declined from TNM-I to TNM-IV, whereas EBV DNA was not detected in the cases of tumor extension where primary tumor cannot be assessed (Tx). This finding supports earlier reports about the time specific and determined role of EBV oncogenic activity in early phases of UCNT development [35,36], while additional genetic and epigenetic changes of NPC cells might occur after the EBV infection [37].

The fact that the majority of EBV found in Serbian isolates was of type 1 was consistent with the worldwide genotype distribution. A potential association between the genetic disposition of the human populations from different geographical regions and specific EBV genotypes has been suggested in previous studies [7].

Discoveries in *LMP1* gene variability and LMP1 functions could be critical for the definition of EBV responsibility for carcinogenesis mechanism. The presence of four known *LMP1* variants with a dominance of B95-8 and Med, represents the already known European distribution [13]. It is particularly interesting that NPC isolates from this region, for the first time, included China1 and NC, unlike variants discovered from other European NPC isolates [17]. So far, China1 has been found in NPC biopsies from China [38] and central and south Russia, while NC has been found in only one sample from a Russian study [32]. In addition, the newly discovered Srb1 and Srb2 variants might represent UCNT-specific *LMP1* polymorphisms with geographical specificity, especially if the previously published data from Serbian patients with benign diseases were considered [23]. The heterogeneity of *LMP1* sequences contradicts previously reported theories. For example, the consistency in *LMP1* variability, which was presented by China1 found in carcinoma tissues from China, suggested that it could be the result of negative selection against the presence of other variants within the tumor. According to this, variant-specific changes in Human leukocyte antigen (HLA) virus epitopes within LMP1 might enable LMP1 expression in the tumor cells with consequent inability to be recognized by LMP1-specific CD8^+^ cytotoxic T lymphocytes [38]. Moreover, in the same study, the absence of the NC variant within NPC was explained by the inability of the NC to inhibit T-cell proliferation and natural killer cytotoxicity because of unique amino acid substitutions in the LMP1 region (amino acids 34 to 40) responsible for immunosuppressive functions [38]. Despite the opposite findings in the present research, it still could be presumed that differences in signaling and biological properties of the LMP1 variants contribute to differences in pathogenicity. Therefore, the B95-8 and NC variants, which were earlier described as “eliminated variants” by negative immune selection, represent possible predictors for favorable TNM stages (I-III) in the present study. It could be assumed that UCNT pathogenesis associated with LMP1 B95-8 or NC variant activity, did not lead to intracranial extension and/or involvement of cranial nerves, hypopharynx, orbit, etc., or that negative immune selection eliminated these variants except in advanced cancer stages.

Among the different types of *LMP1* gene variability, deletions remain a delicate region of investigation for their specific role in carcinogenesis. Of the 10 LMP1 isolates with deletions, four had the frequently described 30-bp deletion, while six had a rare 69-bp deletion. The generation of the deletions during replication is based on slipped-strand mispairing of two 9-nucleotide repeats coding two identical triplets, laterally positioned from one of the deletions [39]. Therefore, one of the repeats constituted the first or the final nucleotide of the deletions.

To date, there are two relevant concepts of the role of deletions in carcinoma cells. The first is the association with geographical and ethnic-group characteristics, and the other is based on direct impact on the development of carcinoma [33]. There have been discrepant findings of the frequency of *LMP1* deletions in NPC tissue. In Serbian biopsies, the frequency was 28.6%, and in other non-endemic regions for NPC such as Europe, North America, and North Africa, the frequency of *LMP1* deletion was determined to be 55–75% in the biopsies, without differences between isolates from NPC and healthy patients [40]. In spite of the dominance of non-deleted *LMP1* in this study, statistical analysis revealed an important aspect of deletion appearances, since deletions have been identified as a possible risk factor for the most disfavorable TNM stage. Patients with non-deleted *LMP1* almost never had metastasis in lymph nodes at a distance more than 6 cm from the primary tumor, and/or to supraclavicular fossa (stage N3). On the other hand, a deletion in *LMP1* was found in 71% of biopsies representing TNM stage IVB. The idea of associations between *LMP1* deletions and aggressive carcinogenesis has existed for almost two decades. It is known that LMP1 could induce proteins with pro-angiogenic functions such as matrix metalloproteinase 1 and vascular endothelial growth factor [41]. It was particularly shown that key role in the activation of vascular endothelial growth factor and angiogenesis had LMP1 driven induction of cyclooxygenase-2 and hypoxia-inducible factor-1α [42]. Also, there is some indirect evidence in the literature that LMP1 could induce progression of metastasis [43,44]. Subsequently, it has been shown that LMP1 had the capacity for modulation of metastatic property by inducing matrix metalloproteinase 9, upregulating the expression of mucin 1 and ezrin, and downregulating inhibitors of metastasis such as RECK1 [45], and also upregulating tyrosylprotein sulfotransferase 1 and tyrosine sulfation of chemocine receptor 4 [46]. In addition, LMP1 induction of fibronectin by activation of activin A and transforming growth factor beta signalling might also contribute to tumor cell invasiveness [41]. Even though the clinical reports within this field are scarce, a similar assumption was demonstrated by researchers from Thailand where NPC biopsies of TNM stages III-IV had 21 times more deleted *LMP1* than did NPC biopsies of TNM stages I-II [34]. On the other hand, in Malaysia, a correlation between deletion and metastatic NPC could not be established [33].

The 69-bp deletion is hard to find in NPC. According to previously published data, the prevalence of this deletion varied from 3.3% in NPC isolates found in North Africa [47], to 27% in NPC isolates found in Russia [32]. In isolates from this study, the prevalence of the 69-bp deletion was 17%, similar to results from Russia. As the 69-bp deletion did not correlate with any specific clinical outcome, and was not detected in earlier benign lesions from Serbian patients [23], it could be assumed that the 69-bp deletion might represent a predictive marker for NPC genesis in non-endemic regions such as Serbia and Russia. In North Africa, where the prevalence of the 69-bp deletion is low and non-specific to NPC isolates, nasopharyngeal carcinoma has an intermediate incidence rate of 8–12 per 100 000 persons per year [47].

The amino acids between 322 and 366 in the C terminal region of LMP1 had been described as a mutational hot spot because of numerous substitutions that occurred during the evolution of LMP1 variants [39]. Besides the amino acid substitutions at the seven LMP1 characteristic positions, the most prominent substitutions were Gly→Ser at 212 (65.7%) and Ser→Asn at 309 (68.6%). Identification of additional substitutions unique for single variants might serve as additional markers for discrimination of variants in specific geographical regions.

Although associations between the variability of the *EBNA1* gene and geographical origin were clearly demonstrated, it was not clarified whether nucleotide changes had any significant influence on the development and pathogenesis of tumors. The distribution of EBNA1 subtypes and dominance of P-thr discovered in this study supported previously described European distributions, except for the first identification of V-ala in European NPC [17].

Characterization of each EBNA1 subtype was completed by identification of all subvariants within the scope of one subtype. The most significant variability was demonstrated within P-thr subvariants (P-sv-2, -4, -5, and -6). Of the four new subvariants, the identification of P-thr-sv-5 was particularly significant because this was the largest homogeneous group of EBNA1 isolates wherein a new subvariant was discovered. Although there is no evidence of functional associations between specific EBNA1 variability and direction of pathogenesis, some assumptions have been presented in the literature. For example, there is a theory about the influence of the V-val polymorphism on NPC progression [48]. In addition, there is another theory about the correlation between EBV gene variability with environmental factors and genetic predisposition of the infected host [19]. It follows that P-thr-sv-5 could be a UCNT-specific EBNA1 subvariant and might serve as a specific diagnostic marker for UCNT evolution. It could also be possible that there is a multifactorial influence of P-thr-sv-5 together with genetic predispositions of the Serbian population. It would be essential to investigate the molecular background of progression between P-thr-sv-5 and host cell *in vitro*.

P-ala isolates were identified as subvariants P-ala-sv-1 or P-ala-sv-2, and they have already been demonstrated in isolates evaluated by a Danish study involving patients with NPC and lymphoma and healthy controls [21]. Therefore, they undoubtedly represent European-specific EBNA1 variability [17]. The V-ala subtype is very rare and has only been found in a South American population [26]. Notably, V-ala-sv-1 from this study was identical to the subvariant V-ala-iii, which was described in one Hodgkin lymphoma isolate from Argentina [26].

Since there is a spectrum of diversities within a single EBV gene, different combinations of genome variability could have significance in specific disease characteristics [40]. Only few studies aimed to define EBV polymorphisms between *EBNA2*, *LMP1*, and *EBNA1* genes, and they had been performed in Chinese and Argentine isolates [26,40]. Although in both studies NPC isolates were not included, there was not any correlation between specific polymorphisms and type of disease.

The comparative analysis between different EBV gene variabilities showed for the first time a significant difference in distribution of *LMP1* variants and the presence of *LMP1* deletions between *EBNA1* subtypes. Thus, there were associations between two LMP1 variants and P-thr: China1 (in 100%) and Med (in 83.3%). Moreover, P-ala was encountered in almost all cases together with non-deleted *LMP1* (93.3%). However, the most important was the fact that those associations had shown correlation with TNM tumor progression. Firstly, both EBNA2 genotypes in combination with B95-8 or NC had never been present in TNM stage IVB. Similarly, the combination between B95-8 and NC and any other EBNA1 subtype, as well as combinations of non-deleted LMP1 and any other EBNA1 subtype. Considering these results, it is clear that *LMP1* deletions have the crucial role in cancer progression to stage N3, independently of *EBNA2* or *EBNA1* variability. Finally, we have identified a possible risk factor for TNM stage IVB: *type 1/Med/P-thr*, a specific combination of *EBNA2* genotype/*LMP1* variant/*EBNA1* subtype. This combination was found in one third of UCNT isolates.

NPC is distinguished from other carcinomas of the head and neck by its epidemiology, histopathology, clinical characteristics, and therapy. EBV findings in this study asserted that EBV activity has a time-specific and determined role in early UCNT oncogenesis. *LMP1* variability showed four known and two new variants, with the first detection of China1 and NC variants in European NPC. New variants Srb1 and Srb2 might represent UCNT-specific LMP1 polymorphisms. Concerning differences in pathogenicity, variants B95-8 and NC represented possible predictors for favorable TNM stages. On the other hand, *LMP1* deletions, the 30-bp deletion and the 69-bp deletion, have been identified as possible risk factors for the most disfavorable TNM stage, independent of *EBNA2* or *EBNA1* variability. In addition, this study identified for the first time a possible risk factor for stage N3 in a specific combination of variability of three EBV genes: *type 1/Med/P-thr*. Of four new EBNA1 subvariants, P-thr-sv-5 revealed a potential diagnostic significance for UCNT evolution. All associations discovered require advanced molecular investigations in order to analyze the mechanisms of their generation and circumstantial influences on host cells, especially because of their significance in UCNT pathogenesis.
